# Adenoid basal carcinoma of the uterine cervix: a case report

**DOI:** 10.3389/fonc.2025.1641219

**Published:** 2026-01-06

**Authors:** Yuan Tan, Liqin Lai

**Affiliations:** Department of Pathology, Tongde Hospital of Zhejiang Province, Hangzhou, China

**Keywords:** adenoid basal carcinoma, adenoid cystic carcinoma, basal squamous cell carcinoma, case report, immunohistochemistry

## Abstract

**Background:**

Adenoid basal carcinoma (ABC) is a rare human papillomavirus (HPV)-related malignancy of the uterine cervix that predominantly affects postmenopausal women, and it is a low-grade tumor with a favorable prognosis. This report presents a case of ABC along with a detailed immunohistochemical analysis.

**Case presentation:**

A 65-year-old woman tested positive for HPV types 16, 35, and 56 during routine screening. A subsequent cervical biopsy indicated adenocarcinoma. The patient was asymptomatic. She underwent radical hysterectomy, bilateral salpingo-oophorectomy, and bilateral pelvic lymph node dissection. A comprehensive pathological examination of the hysterectomy specimen confirmed ABC with a focal component of high-grade squamous intraepithelial lesion (HSIL). The postoperative course was uneventful, and no recurrence or metastasis was observed during 4 years of follow-up.

**Conclusion:**

Adequate tissue sampling and appropriate immunohistochemical profiling are valuable for accurate diagnosis and differentiation of ABC from other malignant cervical lesions.

## Background

Adenoid basal carcinoma (ABC) is an extremely rare malignant epithelial neoplasm, accounting for less than 1% of all cervical carcinomas. It predominantly occurs in postmenopausal women, with a mean age of 64.6 years (1, 2), though rare cases have been reported in patients around 20 years of age (3). Patients with pure ABC are usually asymptomatic due to its indolent clinical course, and the cervix typically appears grossly normal. Consequently, most cases are incidentally detected during procedures such as cervical loop electrosurgical excision (LEEP), conization, or radical hysterectomy. It commonly has a favorable prognosis, and metastasis or recurrence seldom occurs after surgery. Given its clinicopathological resemblance to benign lesions, some experts have proposed the alternative designation “adenoid basal epithelioma” (4). ABC shares a common origin with adenoid cystic carcinoma (ACC), basaloid squamous cell carcinoma (BSCC), and adenoid basal hyperplasia (ABH). Among these, ACC and BSCC are highly invasive, high-grade malignancies with poor prognosis, whereas ABH represents a superficial proliferation of pure adenoid cells (5). Histologically, these entities can be challenging to distinguish from ABC, rendering differential diagnosis clinically significant. Immunohistochemistry (IHC) currently serves as the principal auxiliary tool for differentiation and diagnosis. In recent years, pure ABC has rarely been documented in the world literature, and insufficient familiarity with its histologic features may lead to misdiagnosis, particularly among less experienced pathologists.

## Case presentation

**Figure 1:**
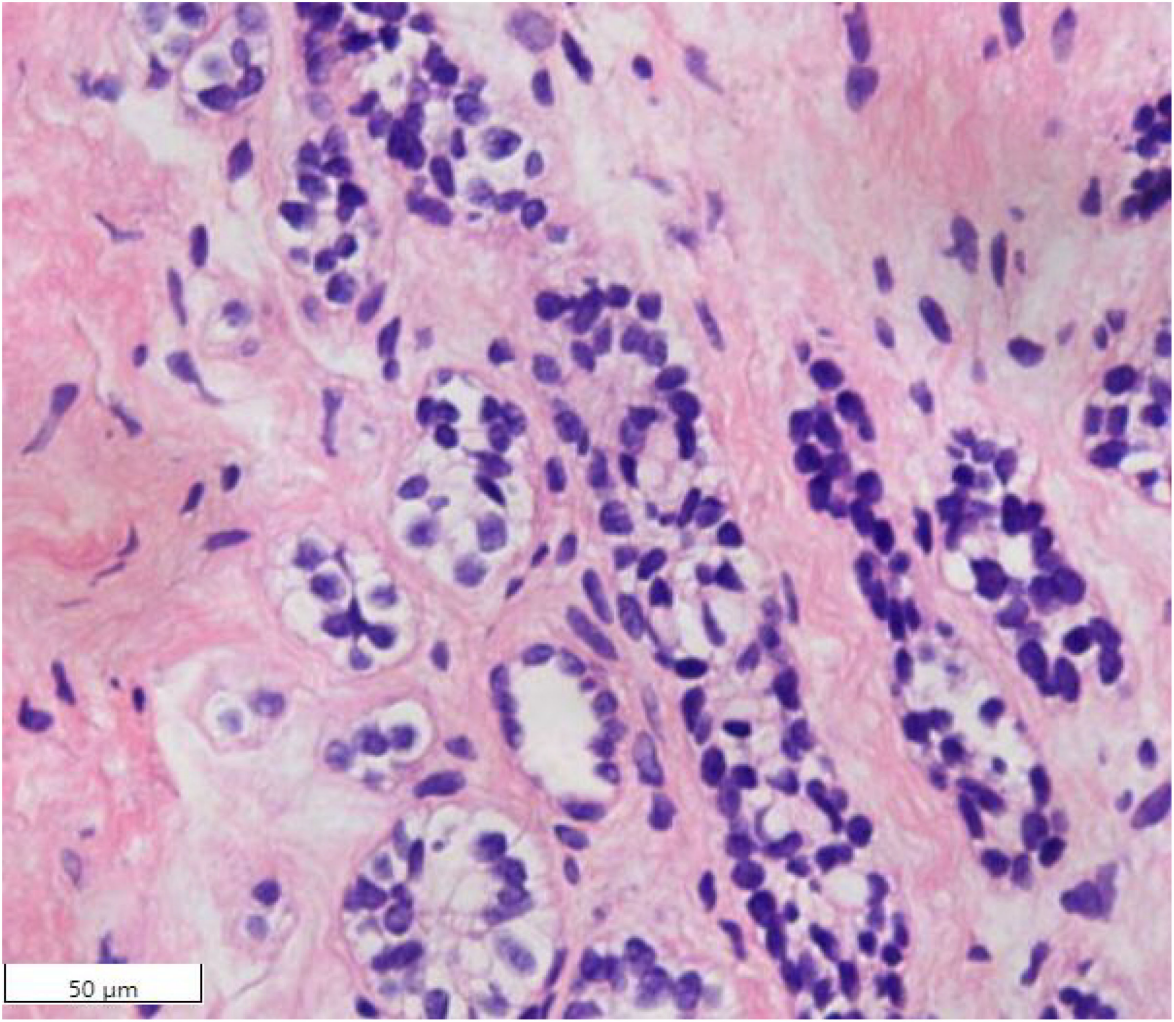
Initial cervical biopsy: neoplastic cells arranged in nests, glands, and acini; glandular lumina are conspicuous (HE, ×20).

A 65-year-old postmenopausal woman (menopausal duration: 20 years) presented for routine cervical cancer screening at a local county-level hospital on October 7, 2020. High-risk human papillomavirus (HPV) testing was positive for types 16, 35, and 56. A subsequent colposcopy with biopsy was performed, and the histopathological examination revealed features consistent with cervical adenocarcinoma. For further confirmation, the biopsy slides were reviewed at a second institution, which also supported the diagnosis of cervical adenocarcinoma. Microscopic examination demonstrated tumor cells exhibiting enlarged nuclei, scant cytoplasm, and hyperchromatic staining. These cells were arranged in nests, glandular tubules, and acinar structures, without significant desmoplastic stromal reaction. Well-defined glandular formations were observed (**Figure 1**). Due to the insufficient biopsy material, immunohistochemical profiling for further subtyping was not performed. Based on the available histomorphological features within the limited tissue sample, a definitive diagnosis of cervical adenocarcinoma was rendered by the local institution. The patient was clinically asymptomatic, and physical examination revealed no notable abnormalities. Gynecological assessment indicated cervical atrophy with a smooth surface.

**Figure 2:**
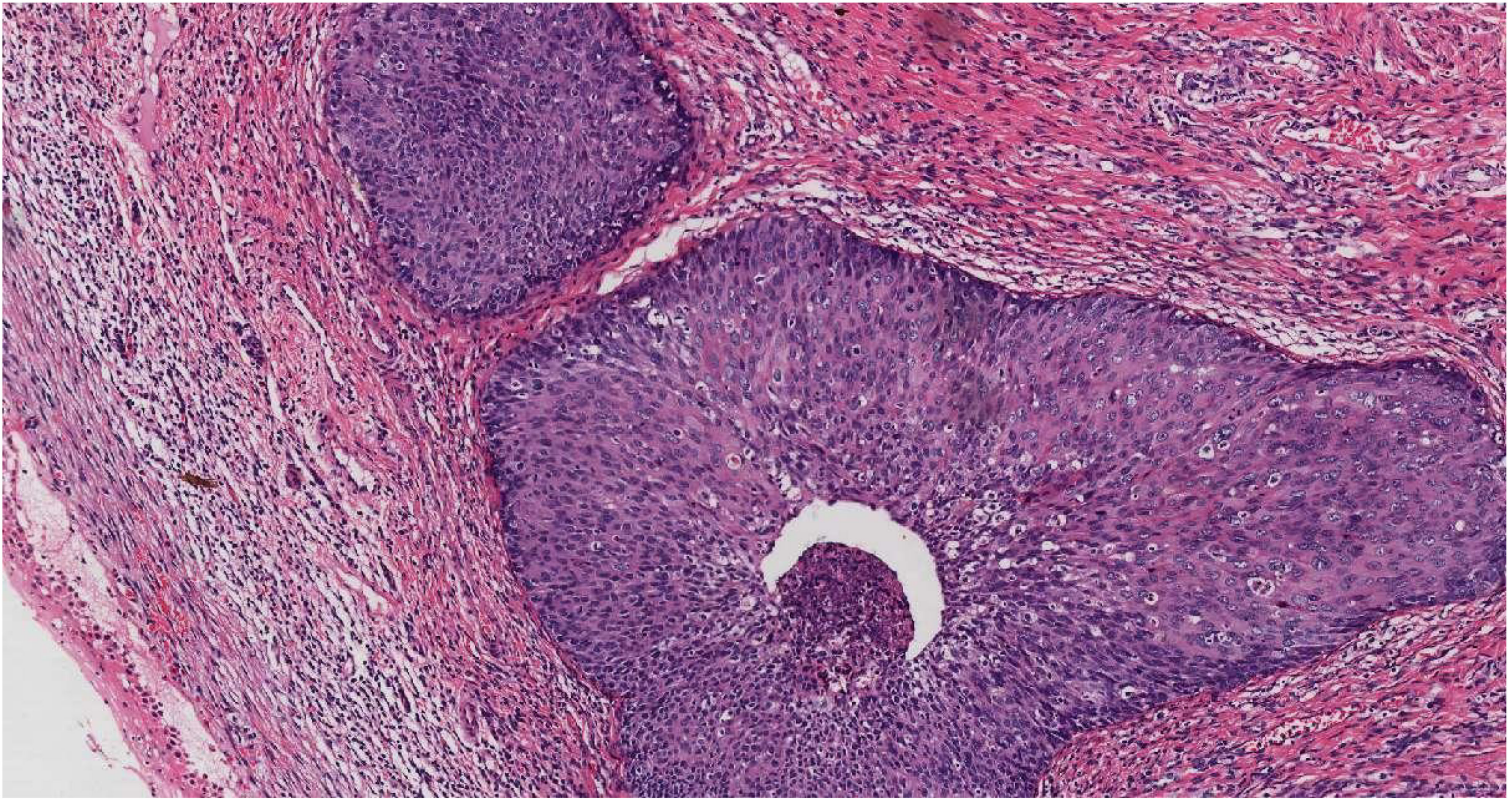
HSIL in the overlying epithelium of the cervix (HE, ×10).

**Figure 3:**
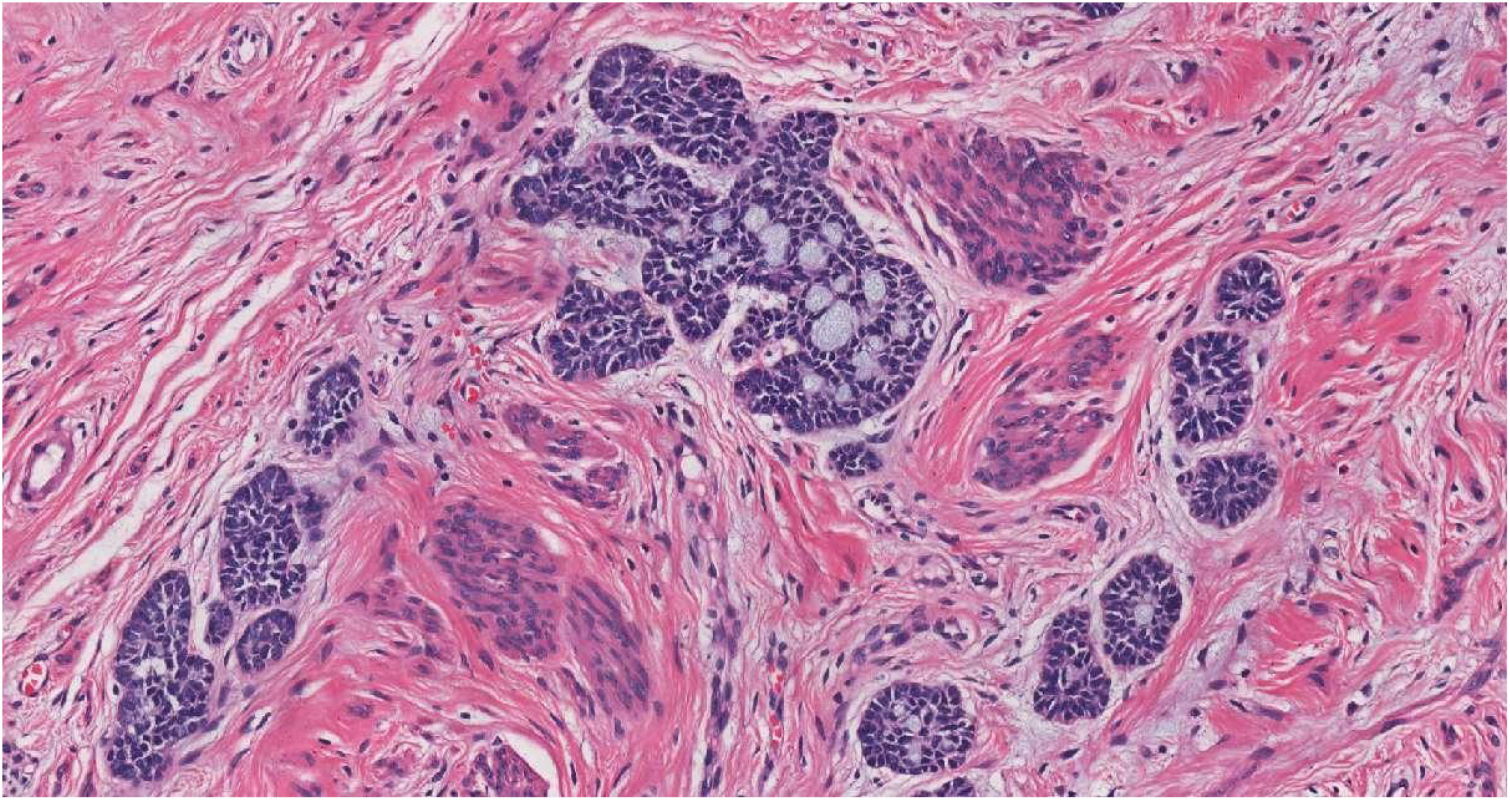
The tumor cells were arranged in nests, with adenoid differentiation and sieve-like structures centrally, and surrounded by palisading cells (HE, ×20).

**Figure 4:**
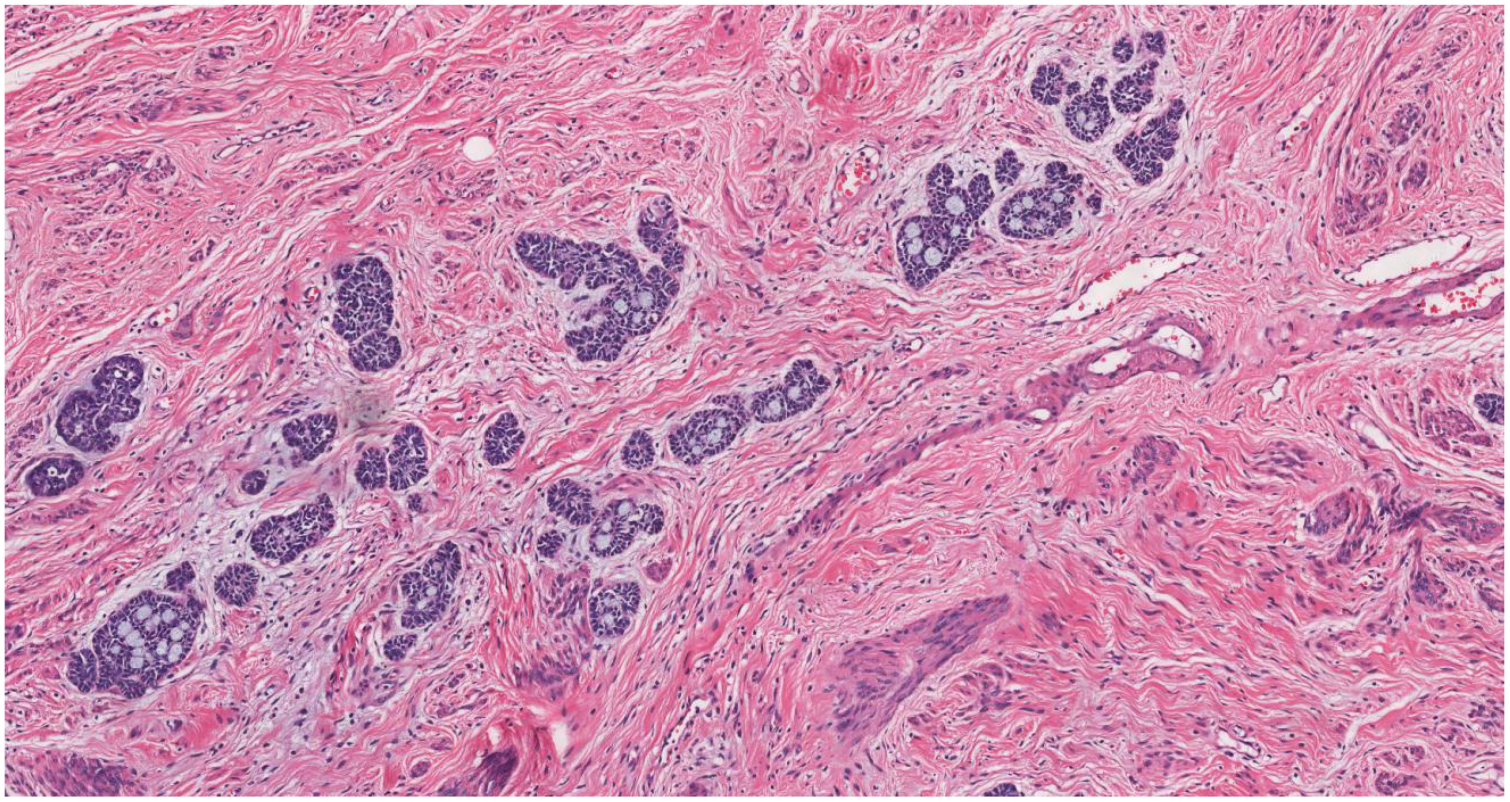
The carcinoma forms small, rounded, oval, or elongated nests with subtle lobulation (HE, ×10).

**Figure 5:**
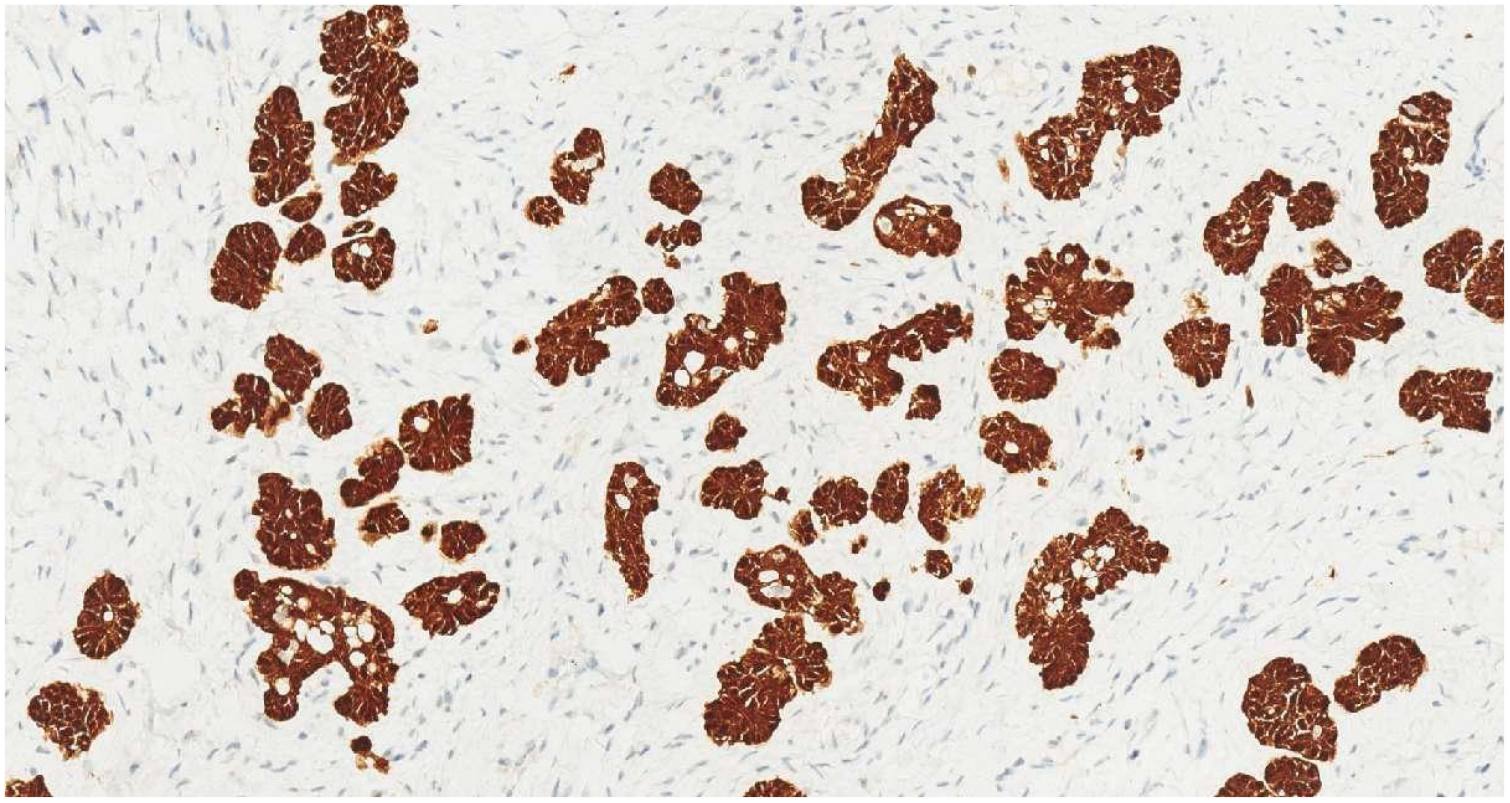
Diffuse, strong nuclear and cytoplasmic p16 expression in tumor cells (IHC, ×20).

**Figure 6:**
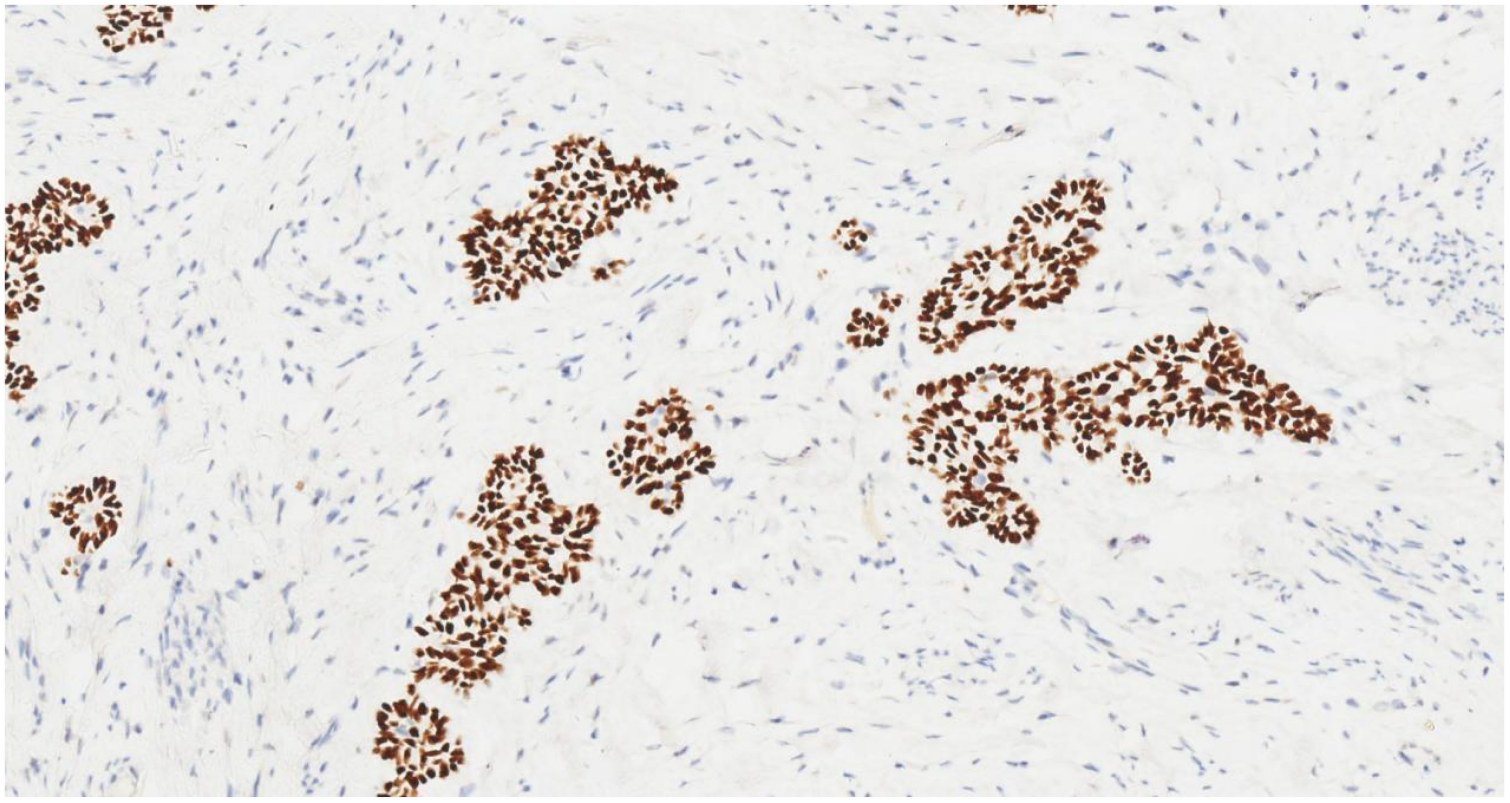
Tumor cells show strong nuclear p63 positivity (IHC, ×20).

**Figure 7:**
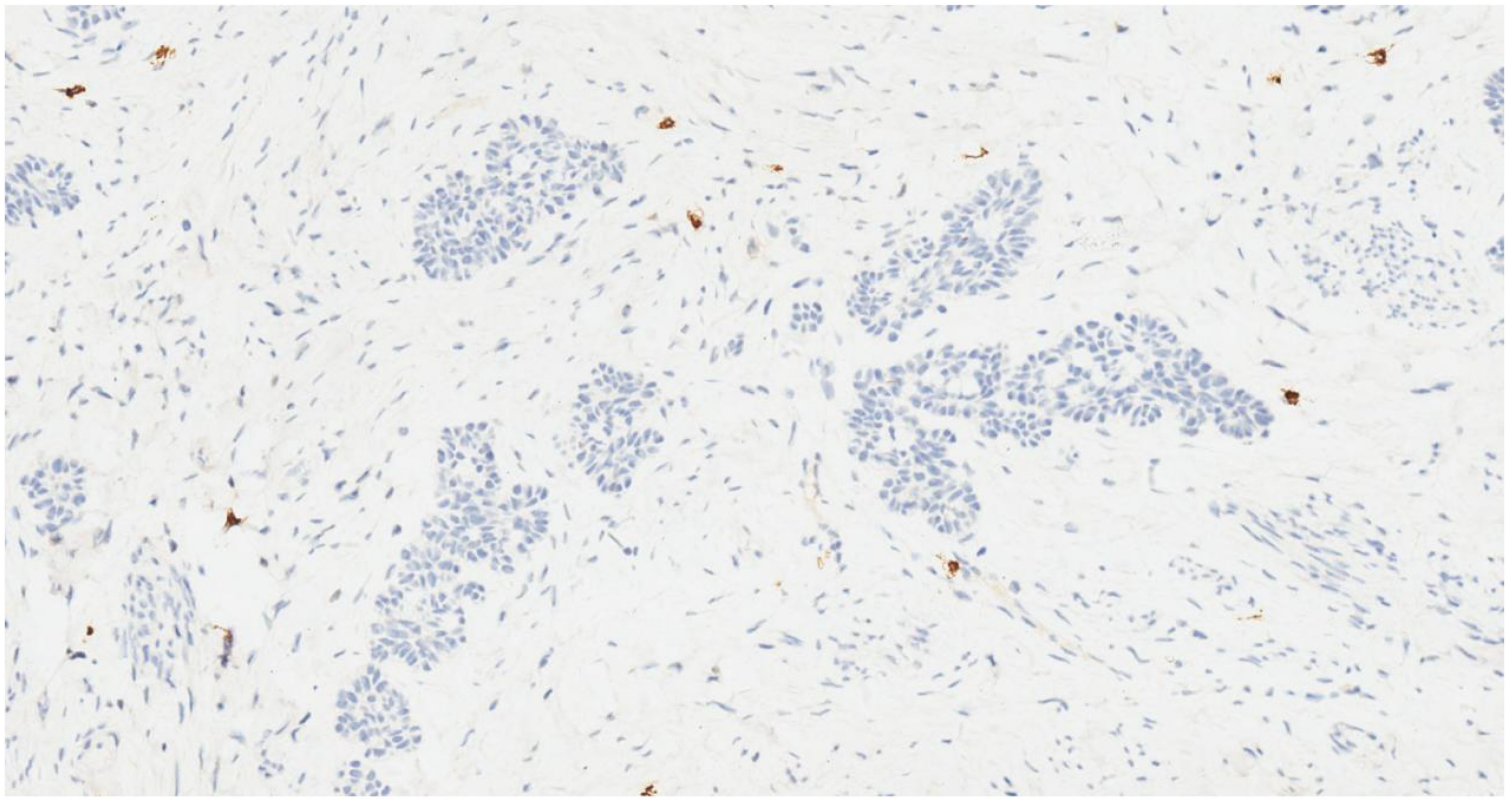
Tumor cells are negative for CD117 (IHC, ×20).

**Figure 8:**
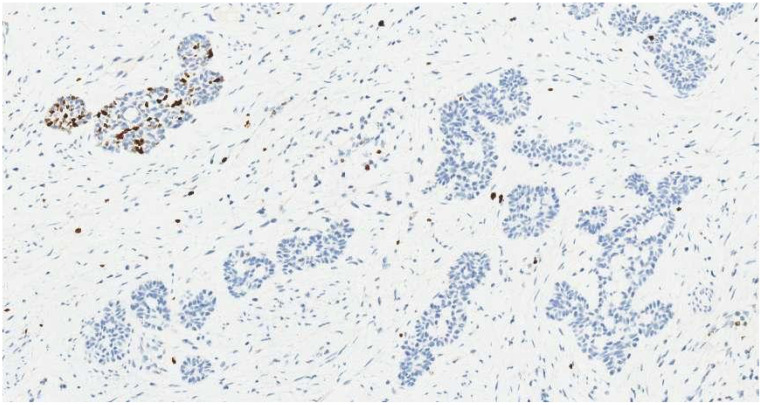
Ki-67 labeling index is approximately 5% in tumor cells (IHC, ×20).

Prominent vasculature was observed on the lower cervical lip, accompanied by vulva atrophy; the vaginal walls were within normal limits. Her surgical history included a laparoscopic appendectomy for an appendiceal abscess in January 2020, and she reported no family history of cervical cancer. Despite the absence of overt symptomatology, the preliminary histopathological diagnosis of cervical adenocarcinoma elicited considerable patient anxiety and apprehension regarding possible malignancy or future progression. The patient expressed a strong preference for expedited therapeutic intervention. Accordingly, she presented to our provincial tertiary hospital on November 3, 2020 for further diagnostic evaluation and definitive management. MRI (non-contrast and contrast-enhanced) of the abdomen and pelvis showed discontinuous cervical mucosa with intense, patchy enhancement separated by linear low-signal areas. The bladder was unremarkable, and the rectal lumen was mildly dilated. No pelvic lymphadenopathy was identified. In November 2020, the patient underwent radical hysterectomy at our hospital. The resected uterus including the cervix measured 10 × 7 × 5 cm, with endometrial thickness ranging from 0.1 to 0.2 cm. The cervical canal measured 3.5 cm in length, and the mucosal surface at external os was generally smooth but focally irregular. A polypoid lesion measuring 2 × 1.5 cm was identified within the cervical canal. On sectioning, the lesion appeared gray-white, solid, and of firm consistency. Multifocal infiltrating carcinoma was identified throughout all cervical sections, involving approximately one-third of the cervical stromal depth. Adjacent high-grade squamous intraepithelial lesion (HSIL) with gland involvement was observed locally (**Figure 2**). The tumor exhibited a predominantly infiltrative growth pattern characterized by small nests and cord-like structures. The neoplastic nests were relatively small, displaying a variable morphology including round, oval, and elongated configurations with subtle lobulation. Central glandular differentiation with cribriform architecture was observed (**Figure 3**). Cytomorphologically, the tumor comprised well-differentiated basaloid cells demonstrating uniform nuclear sizes, scant cytoplasm, hyperchromatic nuclei, inconspicuous nucleoli, and minimal mitotic activity (**Figure 4**). The surrounding stroma showed either no desmoplastic response or only mild reactive change. No lymphovascular or perineural invasion was identified. Immunohistochemically, the tumor cells showed diffuse positivity for p16 (**Figure 5**), p63 (**Figure 6**), pan-cytokeratin, CK5/6, Bcl-2, and wild-type p53. Focal expression of CK7 and ER was observed. The stains for CD117 (**Figure 7**), S-100, SMA, CEA, vimentin, PR, MUC-1, CD10, chromogranin A, CD56, and synaptophysin were negative. The Ki-67 proliferative index was approximately 5% (**Figure 8**). Pelvic lymphadenectomy identified three left-sided and six right-sided lymph nodes, all negative for metastatic carcinoma (0/9). The pathologic diagnosis was ABC. Morphology and immunohistochemistry established a diagnosis of cervical ABC (AJCC 8th edition: pT1b1 N0). Adenoid basal carcinoma typically follows an indolent course. For stage IB1 lesions with negative surgical margins and no lymphovascular space invasion (as in this case), the 5-year disease-specific survival exceeds 95%. Accordingly, no adjuvant radiotherapy or chemotherapy was administered. The patient remains clinically well, without evidence of recurrence or metastasis at 4 years of follow-up.

## Discussion

In the 2020 World Health Organization (WHO) classification of female genital tumors, ABC is categorized under “other epithelial tumors”—a group that also includes ACC, adenosquamous carcinoma, neuroendocrine tumors, and undifferentiated carcinoma (2). ABC is consistently associated with HSIL and high-risk HPV, most frequently HPV 16 (6). This association is supported by the diffuse immunohistochemical expression of p16 in tumor cells, as reported in the literature (7) and further corroborated in the present case. Clinically, most patients with ABC are asymptomatic or present only with nonspecific symptoms, such as vaginal bleeding, watery discharge, or malodorous leucorrhea. Gynecological examination and colposcopy often reveal no distinct cervical mass, and the findings may be limited to erosion or an otherwise unremarkable appearance. Consequently, a definitive preoperative diagnosis of ABC is seldom established. In the present case, the patient was initially diagnosed with cervical adenocarcinoma based on cervical biopsy findings. However, the final pathological diagnosis following radical hysterectomy revealed ABC. This case illustrates that adequate tissue sampling not only enhances diagnostic accuracy but also enables clinicians to exclude other potential malignant lesions, thereby facilitating the development of more appropriate treatment strategies and improving prognosis assessment. The precise histological origin of ABC remains unclear. Current consensus suggests that ABC likely arises from pluripotent reserve cells of the cervical transformation zone. These cells are considered multipotent, possessing the capacity to differentiate toward both squamous and glandular lineages (1). Several studies have provided support for this hypothesis through both ultrastructure analysis of tumor cells and immunohistochemical marker expression profiles. In the present case, the tumor cells demonstrated immunoreactivity for CK5/6, p63, and Bcl-2, findings that are consistent with previously reported immunophenotypic characteristics of this neoplasm. Microscopically, the tumor was composed of uniform, small basaloid cells arranged in rounded or lobular configurations. The cells at the periphery of the nests typically exhibited a palisading arrangement, with some nests displaying glandular or cribriform architecture. The central areas of the nests frequently showed distinct glandular or squamous differentiation, with occasional foci demonstrating transitional cell-like features. The tumor cells typically infiltrated the superficial layer of the cervical stroma, eliciting minimal stromal reaction. However, focal stromal edema and chronic inflammatory cell infiltration were observed around some tumor nests (8). The histopathological findings in our case were entirely consistent with ABC. ABC represents a distinctive malignant neoplasm whose recognition is clinically crucial given its relatively bland morphology and indolent biological behavior, which contrasts sharply with other basaloid-pattern malignancies. ABH refers to pure adenoid basal proliferations characterized by small, superficial lesions that typically extend less than 0.5 mm into the cervical stroma. Both the size of basaloid nests and the depth of stromal invasion serve as key morphological parameters to distinguish ABH (5). Unlike ABC, ABH shows no association with HPV infection, rarely demonstrates squamous differentiation, and consistently exhibits negative p16 immunoreactivity, whereas ABC typically displays the opposite pattern. Additionally, an elevated Ki-67 labeling index provides further evidence supporting a diagnosis of ABC over ABH. ACC represents the most challenging differential diagnosis for ABC, as both entities share morphological features within the same basaloid spectrum. However, ACC exhibits a significantly more aggressive clinical behavior, and patients frequently present with postmenopausal bleeding as the presenting symptom. Gross examination typically reveals polypoid masses. Microscopically, tumor cells demonstrate characteristic atypical cribriform and solid sheet-like architectural patterns. The cystic lumina contain distinctive basement membrane-like material, exhibiting frequent cellular atypia and mitotic activity, accompanied by a prominent stromal reaction (9). Immunohistochemically, classic ABC exhibits diffuse positivity for broad-spectrum cytokeratin, p63, and p16, whereas CD117 expression is absent or weakly focally positive (10). In contrast, ACC demonstrates positive staining for cytokeratin, CEA, and EMA and typically shows strong CD117 immunoreactivity (3, 10). CD117 expression status represents the most valuable immunohistochemical parameter to distinguish between these two lesions. In our present case, ABC exhibited the expected CD117 negativity. Additionally, NKX3.1 shows selective expression in the glandular components of ABC lesions but remains negative in ACC components, providing another useful diagnostic marker for this differential diagnosis (11). BSCC represents an uncommon and aggressive variant of squamous cell carcinoma with different biological and morphological characteristics. BSCC typically exhibits irregular tumor nest contours with frequent central necrotic zones, marked cellular atypia, and readily identifiable mitotic figures (12). Conversely, ABC demonstrates smaller tumor nests with a relatively bland cytomorphology. Although prominent squamous differentiation in the central portions of ABC nests may occasionally mimic BSCC morphology, several distinguishing features exist. Liang Y and colleagues demonstrated that loss of CK17 immunoreactivity within squamous differentiation component and a lower Ki-67 proliferation index in basaloid cell components are diagnostically useful features favoring ABC over BSCC (13). These immunohistochemical findings, combined with the characteristic architectural patterns and cytomorphologic features, enable a reliable differentiation between these two entities with significantly different clinical behaviors and prognostic implications. ABC exhibits indolent biological behavior with benign-like characteristics and is associated with an excellent prognosis; consequently, conservative management may be appropriate in most cases. However, in the present case, although the patient was clinically asymptomatic, the initial endocervical biopsy unequivocally established a diagnosis of invasive cervical adenocarcinoma. Furthermore, concurrent infection with high-risk HPV subtypes 16, 35, and 56 was documented, suggesting a substantial malignant potential. The patient also reported significant psychological distress and explicitly requested definitive surgical intervention. Accordingly, radical hysterectomy was performed based on her individual clinical circumstances. Although the final histopathologic diagnosis revealed ABC, this surgical approach achieved complete excision of the lesion, eliminated the risk of residual disease, and minimized the potential for recurrence. In this clinical context, the procedure represented an optimal therapeutic option that addressed both the medical and psychological needs of this particular patient.

## Conclusion

ABC may occur as a pure neoplasm or in combination with other malignant types of cervical cancer. In mixed tumors, the clinical prognosis and treatment are primarily determined by the most aggressive component present. Therefore, it is critical to exclude other high-grade malignancies. Adequate tissue sampling is essential to identify or rule out associated high-grade components. In diagnostically challenging cases, a targeted immunohistochemical panel can aid in accurate recognition and differentiation from other malignant cervical lesions.

## Data Availability

The original contributions presented in the study are included in the article/supplementary material. Further inquiries can be directed to the corresponding author.
